# Alpine Glacier Change in the Eastern Altun Mountains of Northwest China during 1972-2010

**DOI:** 10.1371/journal.pone.0117262

**Published:** 2015-02-27

**Authors:** Xinyang Yu, Changhe Lu

**Affiliations:** 1 Key Laboratory of Land Surface Pattern and Simulation, Institute of Geographical Sciences and Natural Resources Research, Chinese Academy of Sciences, Beijing, China; 2 University of Chinese Academy of Sciences, Beijing, China; Institute of Tibetan Plateau Research, CHINA

## Abstract

Accurately mapping and monitoring glacier changes over decades is important for providing information to support sustainable use of water resource in arid regions of northwest China. Since 1970, glaciers in the Eastern Altun Mountains showed remarkable recession. Further study is indispensable to indicate the extent and amplitude of glacial change at basin and individual glacier scale. In this study, spatiotemporal glacier changes referring to the year 1972, 1990, 2000 and 2010 were studied for the Eastern Altun Mountains using Landsat MSS/TM/ETM+ images and glacier volume-area scaling. The results demonstrated that the total area and volume of glaciers in EAMs decreased significantly by 10.70±0.57 km² (19.56±10.41%) and 0.61±0.03 km³ (23.19±11.40%) during 1972–2010, respectively. More than half of the total receding area occurred during 1990–2000, primarily due to higher temperature increasing. However, varied response of individual glaciers indicated that glacier change was also affected by glacier dynamics, which was related to local topography. In addition, five glaciers unrecorded in the glacier inventory of China were reported in this study.

## Introduction

Alpine glacier, which is known as mountain glacier, has long been considered one of the most visible indicators of climate change [1]. As the main water source of rivers in arid regions, northwest China, alpine glaciers are essential to support the sustainable development of oases [2]. Since 1970s, increasing availability of remote sensing data has allowed for cost-effective estimation and assessment of spatiotemporal variation of glaciers over decades [3–5]. Recent studies in the Qilian Mountains [6, 7], Tarim basin [8] and Tianshan Mountains [2, 4] of northwest China have reported prominent glacier shrinkage during past decades. Compared to glacier surface area, changes in glacier thickness and volume have more important and direct impacts on potential runoff of rivers. Traditional methods for glacier thickness measuring, e.g. snow pits or ground penetrating radar (GPR) measurement, are difficult to accomplish for a large amount of glaciers due to its high costs and risks in field surveys. Recently, the Shuttle Radar Topography Mission (SRTM) and Geoscience Laser Altimeter System (GLAS) data carried onboard the Ice, Cloud, and Land Elevation Satellite (ICESat) were selected as source data in various studies for ice thickness assessment [9–11]. However, for regions without high quality topographical maps coverage, great uncertainty existed in the glacier thickness acquisition, and thus area-volume scaling became a feasible alternative to assess the glacier volume variation at regional scales [7, 12].

**Fig 1:**
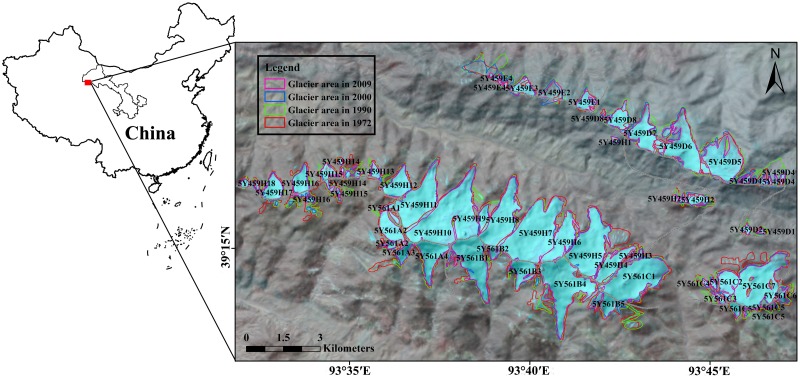
Location of the study area. The map on the right represents the outlines of glaciers in 1972, 1990, 2000 and 2010.

The Eastern Altun Mountains (EAMs) are located on the northern edge of the Tibet Plateau, between western Gansu and Qinghai provinces in northwest China (Fig. 1). Since the glacier inventory of China was compiled in 1981 based on data investigated during 1956–1970s, surface area of glaciers in EAMs receded significantly [13]; however, no further glacial research has been done in glacier change patterns and volume variation at basin and individual glacier scale. In this study, a glacier inventory of glacier surface area in EAMs for the year of 1972, 1990, 2000 and 2010 was established based on Landsat MSS/TM/ETM+ images, and the glacier volume was estimated using area-volume scaling method. Moreover, the difference between different basins and individual glaciers were compared, and the relationship between glacier change and climate variation were analyzed.

## Materials and Methods

### Ethics Statement

Landsat images used in this study were downloaded from the United States Geological Survey (USGS, http://glovis.usgs.gov) and climate data were obtained from the China Meteorological Data Sharing Service System (CMDSSS, http://cdc.cma.gov.cn/home.do). This field study did not involve any endangered or protected species, and no specific permissions were required for EAMs.

### Study area

EAMs cover an area of​​ 344.17 km^2^ with an altitude from 5,002 to 5,828 m above sea level. It has a cold arid climate with annual average temperature between -1.66 and 0.99°C and annual precipitation between 20 and 110 mm. According to the glacier inventory of China, EAMs have 44 glaciers [14], distributed in two basins: Cuimutugou basin and Suganhu basin. Cuimutugou basin (CN5Y459) is the source of the Danghe River covering 28 glaciers in three sub-basins, Qiligou (5Y459D), Liuchengzigou (5Y459E) and Qingshigou (5Y459H). Suganhu basin (CN5Y561) is the source region of the Haertenghe River with 16 glaciers in three sub-basins: Jiaermasayi-Lenghu, Jiaermasayi and Wuminggou (Table 1). For glaciers in EAMs are relatively small in area [14] and more sensitive to climate change. Therefore, EAMs are a representative region to study glacier variation and its relationship to climate change.

**Table 1:** Glacier variation in EAMs, northwest China.

Basin	Sub-basin	Glacier ID	Glacier number
Cuimutugou		**CN5Y459**	**28**
	Qiligou	5Y459D	8
	Liuchengzigou	5Y459E	3
	Qingshigou	5Y459H	17
Suganhu		**CN5Y561**	**16**
	Jiaermasayi- Lenghu	5Y561A	4
	Jiaermasayi	5Y561B	5
	Wuminggou	5Y561C	7

### Glacier outlines delineation

Glacier outlines were delineated by visual interpretation of Landsat images in 1972, 1990, 2000 and 2010 (Table 2). These images were downloaded from the United States Geological Survey (USGS, http://glovis.usgs.gov), and rectified to a UTM projection (Zone 48N, Datum WGS-84) using the nearest neighborhood resampling technique, based on 45 evenly distributed ground control points. After rectification, all the images had a registration error of less than 0.5 pixels. In addition, the glacier inventory of China (1981) and glacier map delineated by field surveys from 1956 to 1970s [14] were collected as auxiliary data.

**Table 2:** Metadata of Landsat images covering EAMs.

Sensor	Date (mm-dd-yyyy)	Resolution (m)
MSS	09–30–1972	60
TM	08–29–1990	30
ETM+	08–21–2000	15/30
TM	08–30–2010	30

Glacier outlines were extracted by visual interpretation of Landsat images three times to minimize the uncertainty of glacier area estimating, and then further processed using spatial analysis tools of ArcGIS 10.1 to detect the glacier change during 1972–2010. The overall accuracy of extracted glacier surface area, which is subjected to the accuracy of geometric registration and image resolutions [15, 16], was examined with the following equations:

$$\begin{array}{l} U_{L}=\sqrt{\sum_{1}^{n}\lambda^{2}}+\sqrt{\sum_{1}^{n}\varepsilon^{2}} \end{array}$$ (1)

$$U_{S}=2U_{L}\sqrt{\sum_{1}^{n}\lambda^{2}}+\sum_{1}^{n}\varepsilon^{2}$$ (2)

where *U*_*L*_ is the accuracy of glacier terminus position, *U*_*S*_ is the accuracy of glacier surface area, *λ* is the resolution of remote sensing images, *ε* is the registration error, and *n* is the total number of images. In this study, image resolutions are 60 m for 1972 and 30 m for other three years, and registration error was 7.50 m. According to the above equations, glacier interpretation error in this study was ± 0.067 km^2^.

### Glacier volume estimation

**Fig 2:**
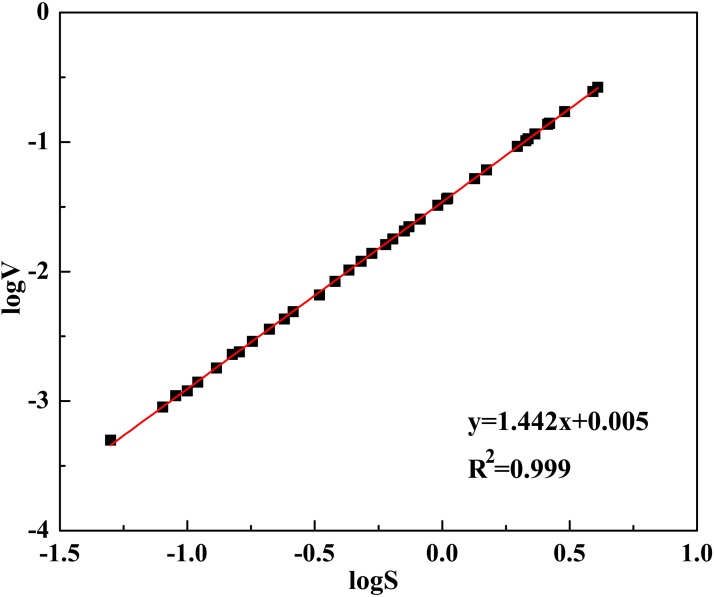
log*V*vs. log*S* for the 44 glaciers in EAMs, northwest China.

Volume for each glacier in EAMs, *V*, was estimated using an exponential function of glacier surface area, *S* [12]:

$$\begin{array}{l} V=kS^{p} \end{array}$$ (3)

where *k* is the constant of glacial mean distribution, and *p* is the scaling coefficient. The coefficients *p* and *k* were determined using the area and volume data of 44 glaciers in the region, which were collected from the inventory of China (1981) on the basis of aero-photographs and field surveys and have an error of less than 5% [14]. To obtain the values for these two coefficients, Equation (3) was firstly transformed into Equation (4), to present the relationship as a linear function of log glacier surface area (log*S*) and log glacier volume (log*V*), and then a linear regression between log*V* and log*S* was performed (Fig. 2). From the results, it was found that the *p* (slope of the fitting line) was 1.442, the constant *p* log *k* was 0.005, and thus *k* was 0.034. This *p* value was close to 1.43, as obtained by Liu et al. [7] in the Western Qilian Mountains of Northwest China, but slightly higher than that of 1.28 to 1.42 obtained in the Alpine Mountains by Chen and Ohmura [18]. The *k* value was same as the results of Bahr [12] and Meier and Bahr [17]. Replacing the coefficients *P* and *K* with the estimated values, Equation (3) was changed as Equation (5), used to estimate the glacier volumes in this study.

$$\begin{array}{l} \log V=p\log S+p\log k \end{array}$$ (4)

$$V=0.034S^{1.442}$$ (5)

## Results

### Glacier change from 1972 to 2010

The glacier distribution and variation in EAMs were mapped in Fig. 1, in which the red line represented glacier polygons in 1972, and green, blue and purple color outlines indicated the glacier scopes in 1990, 2000 and 2010, respectively. Meanwhile, Table 3 summarized glacier change during the study period.

**Table 3:** Area and volume change of glaciers in EAMs during 1972–2010.

Year	Glacier surface area(km^2^)				Glacier Volume(km^3^)	
	Cuimutugou	Suganhu	Total	Change	Total	Change
1972	33.57±0.99	21.13±0.76	54.70±1.25	/	2.63±0.08	/
1990	33.86±0.94	18.3±0.64	52.16±1.14	-2.54±0.27	2.41±0.07	-0.22±0.02
2000	29.78±0.91	16.84±0.64	46.62±1.11	-5.54±0.32	2.14±0.07	-0.27±0.07
2010	28.56±0.92	15.44±0.63	44.00±1.12	-2.62±0.20	2.02±0.07	-0.12±0.01
1972–2010			/	-10.70±0.57	/	-0.61±0.03

In 2010, glacier surface area and volume in EAMs were totaled 44.00±1.12 km^2^ (mean value±standard deviation) and 2.02±0.07 km^3^, respectively. Over the 38 years investigated between 1972 and 2010, the glacier area decreased by 19.56±1.04% and volume by 23.19±1.14%, generally in a state of recession. The receding rate varied during the three study intervals of 1972–1990, 1990–2000 and 2000–2010. The glacier surface area and volume were reduced by 0.26% and 0.46% during 1972–1990, 1.06% and 1.12% during 1990–2000, and 0.56% and 0.59% during 2000–2010, respectively. The most significant recession occurred during the last decade of 20^th^ century, during which the glacier area shrank 5.54±0.32 km^2^, covering more than half of the total retreating area during 1972–2010 (Table 3).

### Regional difference in glacier change

Cuimutugou and Suganhu basins showed different patterns of glacier changes (Table 3). During 1972–2010, glacier area was reduced by 14.92±2.75% in Cuimutugou basin, much lower than that of 26.93±3.31% in Suganhu basin. During 1972–1990, glacier surface area in Cuimutugou basin increased slightly (0.02 km^2^/a), during 1990–2000, it decreased rapidly by 0.41 km^2^/a, and after 2000, it further reduced by 0.12 km^2^/a, displaying a change pattern of slight extension—accelerating recession—decelerating recession. As for Suganhu basin, glaciers displayed an annual retreating rate of 0.16 km^2^/a during 1972–1990, 0.15 km^2^/a during 1990–2000, and 0.14 km^2^/a during 2000–2010, displaying a pattern of decelerating recession through the study period.

**Fig 3:**
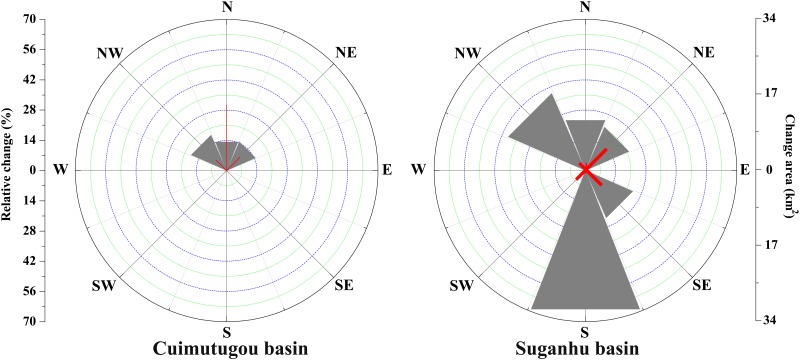
Glacier surface area and the relative change in different aspects of the two basins. Red lines represent glacier surface areas and wedges are the relative change rates in glacier surface area.

The orientation distribution of glaciers in the two basins was much different (Fig. 3). Glaciers in Cuimutugou are northwesterly, northerly and northeasterly oriented, while glaciers in Suganhu basin are distributed in six aspects except the east and west. Further analyses found that glacier retreating rates varied with directions. During 1972–2010, the southerly oriented glaciers showed the maximum retreating rate, receded by 69.49±0.00%, followed by the northwesterly and northerly oriented glaciers, retreated by 39.19±10.62% and 24.78±0.00%, respectively. Glaciers in south aspects had no change occurring during 1972–2010.

Furthermore, glaciers in the study area were divided into four classes by selecting 0.1, 0.5 and 1 km^2^ as thresholds. It was found that the relative reduction of small glaciers was higher than that of large glaciers during 1972–2010 (Table 4), except 4 small glaciers (< 0.1 km^2^) in Cuimutugou. The relative glacier recession rate decreased significantly with increasing glacier size, indicating that small glaciers were more sensitive to climate change than large ones.

**Table 4:** Area changes of glaciers with various size classes in EAMs.

Basin	Glacier size(km^2^)	Number of glaciers	Glacier surface area (km^2^)		Relative change
			1972	2010	(%)
Cuimutugou	<0.1	4	0.29±0.13	0.57±0.25	+96.55±19.20
	0.1–0.5	7	2.35±0.13	1.59±0.56	-32.34±11.23
	0.5–1	6	4.41±0.17	3.00±0.21	-31.97±13.27
	>1	11	26.52±1.19	23.40±1.16	-11.76±24.76
Suganhu	0.1–0.5	5	1.34±0.08	0.56±0.04	-58.21±7.45
	0.5–1	5	3.97±0.14	2.37±0.23	-40.30±14.95
	>1	6	15.82±1.22	12.51±0.92	-20.92±38.09

Although most glaciers in EAMs receded significantly during1972–2010, three glaciers in Cuimutugou basin advanced during 1972–2010 (Table 5). The Glacier 5Y459E2 and Glacier 5Y459H1 extended greatly with the area increased by 4.3 and 1.5 times during the study period, respectively, while Glacier 5Y459H11 expanded slightly by 0.01±0.00 km^2^. This could be due to the effect of local glaciological conditions, such as glacier size, slope, aspect, etc.

**Table 5:** Advanced glaciers in EAMs during 1972–2010.

Basin	Sub-basin	Glacier ID	Latitude	Longitude	Area (km^2^)	
					1972	2010
	Liuchengzigou	5Y459E2	N39°19.16′	E93°39.89′	0.08±0.02	0.43±0.01
Cuimutugou						
	Qingshigou	5Y459H1	N39°18.43′	E93°31.64′	0.02±0.00	0.05±0.01
		5Y459H11	N39°16.32′	E93°36.49′	2.36±0.13	2.37±0.15

### Glaciers unrecorded in the glacier inventory of China

Five glaciers distributed in the west extreme of Liuchengzigou and Qingshigou sub-basins (Table 6) were found unrecorded by the glacier inventory of China. Including these unrecorded glaciers, the EAMs should have a total of 49 glaciers. According to glacial coding rules, these five glaciers can be encoded as Glacier 5Y459E4/5Y459E5/5Y459E6 (Liuchengzigou sub-basin), and Glacier 5Y459H18/5Y459H19 (Qingshigou sub-basin), respectively (Table 6). During the study period, the first three glaciers in Liuchengzigou sub-basin remained almost unchanged in surface area, while the other two retreated 0.01 km^2^ and 0.05 km^2^, respectively.

**Table 6:** Five unrecorded glaciers in EAMs.

Basin	Sub-basin	Code	Latitude	Longitude	Area (km^2^)	
					1972	2010
	Liuchengzigou	5Y459E4	N39°16.432′	E93°31.487′	0.11±0.02	0.11±0.00
		5Y459E5	N39°16.418′	E93°31.847′	0.30±0.01	0.30±0.02
Cuimutugou		5Y459E6	N39°19.253′	E93°37.214′	0.07±0.00	0.07±0.00
	Qingshigou	5Y459H18	N39°19.346′	E93°38.201′	0.09±0.00	0.08±0.02
		5Y459H19	N39°19.444′	E93°37.943′	0.15±0.02	0.10±0.01

## Glacier Change Related to Climate Variation

Among various meteorological factors, temperatures and precipitation are crucial to glacier change [19, 20]. To analyze the effect of climate variation on glacier change, we obtained the annual temperature and precipitation data during the study period by interpolation using the ANUSPLIN method [21], based on weather data from six meteorological stations (Anxi, Dachaidan, Dunhuang, Hongliuhe, Mangya and Lenghu) nearby EAMs, taking into account elevation change.

**Fig 4:**
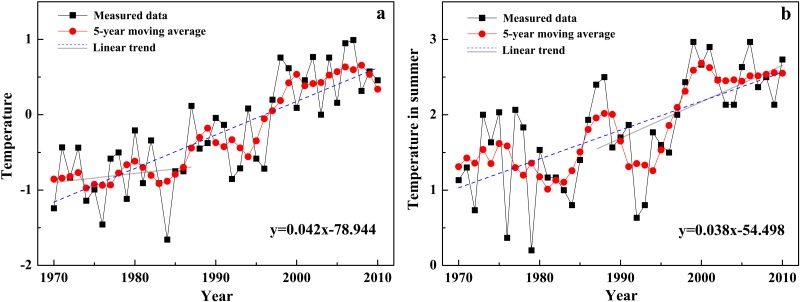
Changes of temperature in EAMs during 1972–2010. (a) variation of annual average temperature (°C), (b) change of summer average temperature (°C).

**Fig 5:**
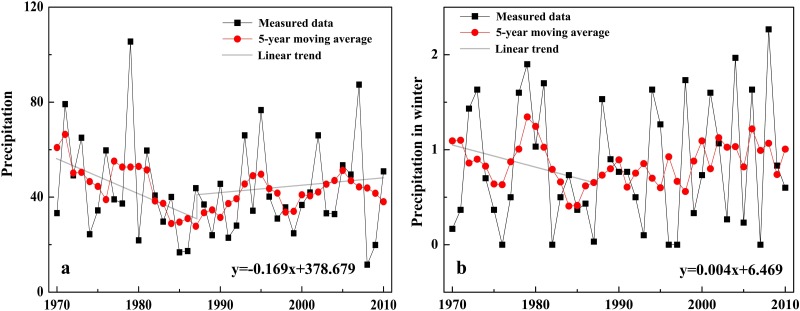
Precipitation variation in EAMs during 1972–2010. (a) change of annual precipitation (mm), and (b) variation of winter precipitation (mm).

During 1972–2010, the annual average temperature in EAMs increased significantly with a rate of 0.42°C/10a (p<0.001, Fig. 4a), which was slightly lower than 0. 43°C/10a in the Western Qilian Mountains (east of EAMs) [7], and higher than 0. 34°C /10a in the Tianshan Mountains in the west [22]. Summer temperature also showed a notable ascending trend of 0.38°C/10a (p<0.001, Fig. 4b), which was consistent with the result of Huang et al. [23]. According to previous studies’ results [16, 24], to offset the mass loss caused by the temperature increase of 0.43°C/10a in EAMs, precipitation in winter should increase at least 0. 36 mm/10a. During 1972–2010, the increase rate of winter precipitation in EAMs was only 0.04 mm/10a, far away to the need of balancing the glacier mass loss due to the temperature rise. In addition, the annual precipitation decreased significantly by 1.69 mm/10a during the same period (Fig. 5). Therefore, temperature increasing was the main factor leading to the glacier recession in EAMs.

Previous study [25] and the Mann-Kendall test [26] results indicated that the year of 1987 was a turning point in the course of annual temperature change in EAMs. From 1972 to 1987, the annual average temperature was significantly increased by 0.16°C/10a (p<0.1, Fig. 4a), and the summer temperature was non-significantly increased by 0.16°C/10a (Fig. 4b); Conversely, the annual and winter precipitation showed significant descending trends of-1.38 mm/10a (p<0.05, Fig. 5a) and -0.23 mm/10a (p<0.05), respectively (Fig. 5b). During the similar period from 1972 to 1990, the annual glacier recession rate was 0.26% per year (Table 3). After 1987, temperature rise was accelerated (but no apparent trend was observed for precipitation), and the glacier recession rate was accordingly increased. From then to 2010, the summer temperature was significantly increased by 0.51°C/10a (p<0.05), 3.2 times of the increasing rate during 1972–1987. Accordingly, the annual glacier recession rate was 0.78%, 3.0 times of that during 1972–1990. Further comparison also indicated that the glacier recession rate was positively related to the temperature increase rate. From Fig. 4, it can be seen that the temperature increase was the quickest during 1990–2000, with the summer temperature increased by 1.41°C/10a. The glacier area showed the greatest recession and the annual recession rate was 1.06%, much higher than that during 1972–1990 (0.26%) and 2000–2010 (0.59%).

For individual glacier, their responses to climate change varied, probably due to their different response times. For instance, the area of Glacier 5Y459H11 expanded by 0.19%/a during 1972–1990, whereas the area of Glacier 5Y459D5 and 5Y561C1 shrank by 0.27 and 1.06%/a, respectively (Table 7). Moreover, the most significant recession of Glacier 5Y459D5 and 5Y561H11 occurred during 1990–2000, while the most prominent retreating for Glacier 5Y561C1 occurred during 1972–1990. This illustrated that except climate factors, glacier changes were affected by other factors, such as glaciological dynamics and microclimate.

**Table 7:** Area changes of three typical glaciers in EAMs, northwest China during the study period.

Basin	Sub-basin	Glacier ID	Year	Area (km^2^)	Relative change (%/a)
Cuimutugou	Qiligou	5Y459D5	1972	2.44±0.16	
			1990	2.32±0.13	-0.27
			2000	2.07±0.07	-1.08
			2010	1.90±0.06	-0.82
	Qingshigou	5Y459H11	1972	2.36±0.13	
			1990	2.44±0.18	+0.19
			2000	2.40±0.15	-0.16
			2010	2.37±0.15	-0.13
Suganhu	Wuminggou	5Y561C1	1972	3.73±0.15	
			1990	3.02±0.11	-1.06
			2000	2.89±0.11	-0.43
			2010	2.90±0.10	+0.04

## Conclusions

Accurate estimation of glacial area and volume has become a highly concerned issue for sustainable development in arid region. By visual interpretation of Landsat images in combination with auxiliary data, we obtained the surface area and volume of glaciers at regional level in EAMs and analyzed their variations from 1972 to 2010. The results indicated that glacier surface area and volume decreased by 19.56±10.41% and 23.19±11.40% during the study period, primarily due to temperature increasing. Glacier change varied greatly among different basins and individual glaciers: the most prominent glacier recession occurred during 1990–2000 and in south aspect; glaciers in response to climate change varied due to glaciological dynamics and microclimate factors. This study confirmed that small glaciers performed more significant change in area than large ones. In addition, five unrecorded glaciers were found and their change characteristics were studied.

Although glaciers in EAMs have generally retreated, it is remarkable that three glaciers advanced during 1972–2010; moreover, the two basins and individual glaciers displayed different change patterns during the study intervals. Due to lack of mass balance and glacier dynamics data, it is not accessible to reveal the change mechanism at glacier scale. Meanwhile, the results may involve some uncertainties, e.g. the glacier volume might be somewhat overestimated by the empirical equation. However, this study presented the change characteristics of glaciers and their responses to climate change in EAMs during the past four decades, which could have a contribution to the understanding of glacier change induced by climate warming in arid region of northwest China.
